# Bacteriemia por estreptococos del grupo *viridans* en niños con leucemia: epidemiología y variables asociadas con la recurrencia

**DOI:** 10.7705/biomedica.8038

**Published:** 2026-04-28

**Authors:** María Emilia Padilla, Guadalupe Pérez, Vanesa Reijtman, Claudia Sarkis, Gabriela Belén González, Lourdes Ferreres, Sandra Gómez, Moira Taicz

**Affiliations:** 1 Servicio de Epidemiología e Infectología, Hospital Nacional de Pediatría S.A.M.I.C. “Profesor Dr. Juan P. Garrahan”, Ciudad Autónoma de Buenos Aires, Argentina Argentina Hospital Nacional de Pediatría S.A.M.I.C. “Profesor Dr. Juan P. Garrahan” Ciudad Autónoma de Buenos Aires Ciudad Autónoma de Buenos Aires; 2 Servicio de Microbiología, Hospital Nacional de Pediatría S.A.M.I.C. “Profesor Dr. Juan P. Garrahan”, Ciudad Autónoma de Buenos Aires, Argentina Argentina Hospital Nacional de Pediatría S.A.M.I.C. “Profesor Dr. Juan P. Garrahan” Ciudad Autónoma de Buenos Aires Ciudad Autónoma de Buenos Aires

**Keywords:** estreptococos del grupo *viridans*, Streptococcus viridans, bacteriemia, leucemia, pediatría, viridans group streptococci, Streptococcus viridans, bacteremia, leukemia, pediatrics

## Abstract

**Introducción.:**

Las infecciones por estreptococos del grupo *viridans* en pacientes oncológicos pueden ser graves, con una gran mortalidad.

**Objetivo.:**

Describir las características clínicas, microbiológicas y evolutivas de los pacientes pediátricos con bacteriemia por estreptococos del grupo *viridans* y leucemia aguda en un hospital de alta complejidad e identificar las variables asociadas con la recurrencia.

**Materiales y métodos.:**

Se trata de un estudio retrospectivo de cohorte. Se incluyeron 108 pacientes de 1 mes hasta 18 años con leucemia aguda y con bacteriemia por estreptococos del grupo *viridans* entre el 2016 y el 2022. Se describieron las características de la cohorte y se hizo un análisis univariado, comparando las características de los pacientes que presentaron un primer o único episodio de bacteriemia con las de aquellos con recurrencia.

**Resultados.:**

Se estudiaron 108 episodios correspondientes a 85 (79 %) pacientes que presentaron un primer o único episodio y a 23 (21 %) que presentaron recurrencias. La mediana de edad fue de 68 meses. Las especies más frecuente fueron *Streptococcus mitis* y *S. oralis* en ambos grupos. Se asoció con recurrencia de la bacteriemia: el uso de antibióticos en los 30 días previos [n = 20 (87 %) vs. n = 52 (61 %), p = 0,03], una mayor duración del tratamiento antibiótico previo, el uso de carbapenem, la gastroenteritis aguda como foco clínico de infección y la etapa de consolidación de la quimioterapia.

**Conclusiones.:**

La bacteriemia por estreptococos del grupo *viridans* predominó en niños con leucemia mieloide aguda. El 21 % fueron recurrencias. La recurrencia se asoció con el uso previo de antibióticos, la gastroenteritis como foco clínico y la etapa de consolidación de la quimioterapia.

## Abstract (en)

**Introduction.:**

Infections caused by *viridans* group streptococci in pediatric oncology patients can be severe and are associated with high mortality.

**Objective.:**

To describe the clinical, microbiological, and outcome characteristics of pediatric patients with *viridans* group streptococci bacteremia and acute leukemia in a high- complexity hospital, and to identify variables associated with recurrence.

**Materials and methods.:**

We conducted a retrospective cohort study including patients aged 1 month to 18 years with acute leukemia and *viridans* group streptococci bacteremia between 2016 and 2022. Patients and episode characteristics were described, and a univariate analysis was performed comparing those with a single bacteremia episode to those with recurrent episodes.

**Results.:**

A total of 108 episodes were identified, of which 85 (79%) were first or isolated episodes and 23 (21%) were recurrences. The median age was 68 months. *Streptoccocus mitis* and *S. oralis* were the most frequently identified species in both groups. Recurrent bacteremia was significantly associated with prior antibiotic use within the preceding 30 days, longer duration of prior antibiotic treatment, use of carbapenems, presence of acute gastroenteritis as the clinical focus of infection, and the consolidation phase of treatment.

**Conclusions.:**

*Viridans* group *streptococci* bacteremia predominantly occurred in children with acute myeloid leukemia. Recurrence accounted for 21% of episodes and was associated with prior antibiotic exposure, gastroenteritis as the clinical focus, and the consolidation phase of chemotherapy.

Las infecciones por estreptococos del grupo *viridans* en pacientes oncológicos se asocian con cuadros graves, con una mortalidad de hasta el 23 % en la población pediátrica ^1^^,^^2^. En la literatura se describen los siguientes factores de riesgo de bacteriemia por este microorganismo: el empleo de citarabina en grandes dosis en el esquema de quimioterapia; la profilaxis antimicrobiana con trimetoprim-sulfametoxazol o fluoroquinolonas; la mucositis grave; los períodos prolongados de neutropenia; la neumonía como foco clínico y la leucemia mieloide aguda como enfermedad de base ^3^^,^^4^.

En ocasiones, puede haber recurrencia de la infección ante los sucesivos ciclos de quimioterapia; sin embargo, las variables asociadas con el desarrollo de recurrencia de la bacteriemia por estreptococos del grupo *viridans* no se encuentran bien caracterizadas.

Así pues, los objetivos del presente trabajo fueron:

1. describir las características clínicas, microbiológicas y evolutivas de los pacientes pediátricos oncológicos con bacteriemia por estreptococos del grupo *viridans* y leucemia aguda en un hospital de alta complejidad, y
2. comparar las características de los pacientes que presentaron el primer episodio de bacteriemia con las de aquellos que presentaron episodios recurrentes, con el fin de identificar cuáles son las variables asociadas con la recurrencia.

## Materiales y métodos

Se realizó un estudio retrospectivo de cohorte en el Hospital de Pediatría J. P. Garrahan, un centro de atención de tercer nivel que cuenta con más de 600 camas de internación, cinco unidades de cuidados intensivos y una unidad de cuidados intensivos neonatales. El hospital recibe pacientes desde el área de emergencias y consultorios externos, tanto por consulta espontánea como remitidos de otras instituciones de todo el país.

Se incluyeron pacientes con edades entre 1 mes y 18 años, con diagnóstico de leucemia aguda, que se encontraban recibiendo quimioterapia y que presentaron, al menos, un episodio de bacteriemia por estreptococos del grupo *viridans* durante el periodo comprendido entre enero del 2016 y julio del 2022.

Se excluyeron aquellos pacientes que no estaban recibiendo quimioterapia al momento de presentar el episodio de bacteriemia.

Se definió «episodio de bacteriemia por estreptococos del grupo *viridans*» como el aislamiento de estas bacterias en, al menos, un hemocultivo obtenido de sangre periférica, de pacientes con leucemia aguda que estaban recibiendo quimioterapia y que presentaban manifestaciones clínicas indicativas de infección sistémica.

Si bien los estreptococos del grupo *viridans* pueden ser colonizadores habituales de la piel y pueden contaminar los hemocultivos, en los pacientes con leucemia aguda bajo quimioterapia con historia clínica de infección, el aislamiento de este microorganismo, incluso en un único hemocultivo, se jerarquiza ^1^.

Se definió «recurrencia de la infección» como la presencia de nuevos hemocultivos positivos para estreptococos del grupo *viridans* luego de un mes de la infección inicial y hasta seis meses después del primer episodio, con aislamiento de la misma especie y con igual perfil de sensibilidad antimicrobiana que el identificado en el episodio inicial.

En función de esta definición, los episodios se clasificaron en dos grupos para el análisis comparativo: 1) primer episodio de bacteriemia por estreptococos del grupo *viridans* y 2) recurrencia.

### Definiciones operativas

- *Tipo de bacteriemia*: se clasificó como primaria cuando no tenía un foco clínico de infección o como secundaria cuando existía un foco clínico de infección inicial.
- *Foco clínico inicial*: signos y síntomas de infección en las primeras 72 horas de internación, que permiten localizarla en algún parénquima específico, catalogado en uno de los siguientes:
- neumonía con distrés respiratorio,
- neumonía sin distrés respiratorio,
- gastroenteritis aguda,
- infección de piel y partes blandas (incluyendo el sitio de salida del catéter venoso central),
- infección respiratoria alta,
- mucositis,
- absceso perianal,
- meningitis o
- tiflitis.
- *Neutropenia*: valor único de neutrófilos menor de 500/mm^3^ o a un valor menor de 1000/mm^3^ con una disminución en los últimos 7 días en relación con la predicción de neutropenia en las 24 a 48 horas siguientes, según las recomendaciones vigentes.
- *Neutropenia profunda*: número de neutrófilos menor de 100 células/mm^3^.
- *Fiebre*: registro único de temperatura axilar ≥ 38,5 °C o dos mediciones de ≥ 38 °C con una separación de, al menos, una hora entre ambas.

### Recopilación de datos

A partir de los datos del Servicio de Microbiología del hospital, fue posible identificar a los pacientes que cumplían con los criterios de inclusión. Se revisaron las historias clínicas y se registraron los siguientes datos: edad, sexo, enfermedad de base, etapa del tratamiento, último esquema de quimioterapia recibido, foco clínico infeccioso inicial, neutropenia, antecedente de haber recibido antibióticos en los 30 días antes de la bacteriemia, antibiótico recibido y duración del tratamiento previo, microorganismo aislado y sensibilidad a antimicrobianos, tratamiento empírico y definitivo, ingreso a la unidad de cuidados intensivos y muerte.

### Aspectos microbiológicos

En cada episodio se tomaron dos muestras de sangre periférica y se inocularon en botellas PF Plus para aislamiento de bacterias aerobias en los pacientes pediátricos. Para aislar las bacterias anaerobias, se usaron las botellas FN plus, donde fueron incubadas durante 5 días en el sistema automatizado BACT/ALERT 3D™ hasta septiembre del 2021.

Posteriormente, se utilizó el sistema BACT/ALERT VIRTUO™. Los cultivos se consideraron positivos ante el desarrollo de estreptococos del grupo *viridans* en, al menos, una de las botellas. Los aislamientos fueron identificados utilizando la espectrometría de masa MALDI-TOF en equipo VITEK MS™ Legacy.

Las pruebas de sensibilidad a la penicilina y a la cefotaxima se realizaron por difusión en placas de Müeller Hinton con el agregado de 5 % de sangre de carnero, con el método epsilométrico, mediante el uso de las tiras reactivas ETEST™.

Todos los productos y equipos usados fueron de la marca bioMérieux^™^ de Marcy L’Etoile, Francia.

Las pruebas se interpretaron según las recomendaciones del *Clinical and Laboratory Standards Institute* (CLSI) del 2022 ^5^.

### Análisis estadístico

Las variables categóricas se expresaron en números absolutos y en porcentajes. Para resumir las variables continuas se utilizaron la mediana y el rango intercuartílico, o la media y la desviación estándar, dependiendo de la distribución de las variables. La normalidad se comprobó en forma gráfica y en forma analítica, mediante la prueba de Wilk-Shapiro. Se compararon las variables de los pacientes en el primer episodio de bacteriemia con aquellas de las recurrencias.

Se realizó un análisis univariado. Para comparar las variables categóricas, se utilizó la prueba de χ^2^; para las continuas, la prueba de Wilcoxon. Se consideró significativo un p <0,05. Se utilizó el *software* Stata™, versión 16.

### Consideraciones éticas

El estudio se desarrolló con base en la normativa nacional e internacional vigente sobre investigaciones en salud y fue aprobado por el comité de ética de la institución. La recopilación y el análisis de los datos se llevaron a cabo respetando la confidencialidad de la información. No se solicitó el consentimiento informado por tratarse de un estudio observacional y retrospectivo.

## Resultados

En el periodo de estudio se practicaron 78 732 hemocultivos, de los cuales 6727 (8,5 %) resultaron positivos. Del total de hemocultivos positivos, en 108 (1,6 %) se aislaron estreptococos del grupo *viridans*.

Se incluyeron 108 episodios de bacteriemia por estreptococos del grupo *viridans* en 85 pacientes y 45 (53 %) eran hombres. Las enfermedades de base fueron: leucemia mieloide aguda en 54 (64 %) pacientes y leucemia linfoblástica aguda en 31 (36 %). Doce (14 %) pacientes se encontraban en recaída de su enfermedad de base. La mediana de edad al momento de la bacteriemia fue de 67,5 meses (rango intercuartílico (RIC): 41 a 104).

Se constató el antecedente de uso de citarabina en 83 (77 %) episodios y el de uso de antibióticos en los 30 días previos en 72 (67 %). Los pacientes presentaban neutropenia al momento de la bacteriemia en 93 (86 %) de los episodios y se documentó fiebre en 101 (93 %).

Sesenta (56 %) episodios fueron casos de bacteriemia primaria, y 48 (44 %) fueron secundarios a un foco clínico de infección inicial. Los focos clínicos más frecuentes fueron la gastroenteritis aguda (n = 14), la mucositis oral (n = 10), la infección de la piel y las partes blandas (n = 11), y la neumonía (n = 5). Un paciente presentó dos focos clínicos de infección.

En 3 (3 %) episodios, los pacientes presentaron sepsis y, en 6 (6 %), choque séptico. Veinticuatro (22 %) pacientes requirieron ingreso a la unidad de cuidados intensivos y 8 (7 %) fallecieron, uno de ellos a causa de la infección por estreptococos del grupo *viridans*. En los cuadros 1 y 2 se muestran las características clínicas y de evolución de los pacientes considerando cada episodio.

**Cuadro 1:** Análisis univariado: características de los pacientes en su primer o único episodio [n = 85 (79 %)] *vs*. en las recurrencias [n = 23 (21 %)] (N = 108)

Variable	Total n (%)			Primer o único episodio n (%)			Recurrencia n (%)			OR (IC_95%_)			p
Sexo femenino	59		(55)	45		(53)	14		(61)	1,70		(0,66-4,40)	0,25
Edad en meses,	67,5		(41-105)	68		(40-110)	66		(47-94)	0,29		(0,98-1,01)	0,83
Inducción	38		(35)	33		(39)	5		(22)	0,44		(0,15-1,29)	0,12
Consolidación	33		(31)	22		(26)	11		(48)	2,66		(1,03-6,90)	0,04
Intensificación	20		(19)	15		(18)	5		(22)	1,29		(0,41-4,04)	0,65
Mantenimiento	3		(3)	2		(2)	1		(4)	1,88		(0,16-21,7)	0,61
Recaída	14		(13)	13		(15)	1		(4)	0,25		(0,03-2,03)	0,16
Presencia de neutropenia <100 células/mm^3^	93		(86)	71		(83)	22		(96)	4,3		(0,53-34,9)	0,10
Uso de citarabina	83		(77)	62		(73)	21		(91)	3,9		(0,8-17,90)	0,08
Uso de antibióticos los 30 días previos	72		(67)	52		(61)	20		(87)	4,23		(1,16-15,30)	0,03
Uso de carbapenem los 30 días previos	22		(20)	14		(16)	8		(35)	2,70		(0,96-7,50)	0,05
Días antibióticos previos, [mediana (RIC)]	4		(1-10)	3		(0-8)	9.5		(5-10)	1,5		(1,20-1,50)	<0,01
Presencia de fiebre	101		(93)	78		(92)	23		(100)			-	-
Sepsis	3		(3)	2		(2)	1		(4)	1,88		(0,16-21,7)	0,60
Choque séptico	6		(6)	5		(6)	1		(4)	0,72		(0,08-6,55)	0,77
Ingreso a la unidad de cuidados intensivos	24		(22)	19		(22)	5		(22)	0,97		(0,31-2,94)	0,94
Muerte	8		(7)	6		(7)	2		(9)	1,25		(0,23-6,6)	0,91

RIC: rango intercuartílico

**Cuadro 2:** Análisis univariado: características de los pacientes en su primer o único episodio [n = 85 (79 %)] *vs*. en las recurrencias [n = 23 (21 %)] (N = 108)

Variable	Total n (%)	Primer o único episodio n (%)	Recurrencia n (%)	OR (IC_95%_)	p
Infección respiratoria alta	2 (2)	2 (2)	0	-	-
Neumonía sin distrés respiratorio	3 (3)	3 (3,5)	0	-	-
Neumonía con distrés respiratorio	2 (2)	2 (2)	0	-	-
Gastroenteritis aguda*	14 (13)	8 (9)	6 (26)	3,39 (1,04-11,10)	0,01
Infección de la piel y de las partes blandas	11 (10)	9 (11)	2 (9)	0,85 (0,16-4,10)	0,79
Mucositis*	10 (10)	8 (9)	2 (8)	0,9 (0,18-4,65)	0,91
Absceso perianal	4 (4)	3 (3,5)	1 (4)	1,24 (0,12-12,5)	0,85
Meningitis	1 (1)	1 (1)	0	-	
Tiflitis	2 (2)	2 (2)	0	-	

* Un paciente presentó los dos focos clínicos señalados.

Todos los pacientes recibieron un tratamiento empírico con un antibiótico betalactámico (cuadro 3). En 51 (47 %) pacientes, se indicó un tratamiento combinado con vancomicina. La mediana de días de tratamiento fue de 10 (RIC: 10-10).

**Cuadro 3:** Antibióticos recibidos en el esquema empírico inicial [n = 85 (79 %)] y definitivo [n = 23 (21 %)] (N = 108)

Variable		Total n (%)	Primer o único episodio n (%)	Recurrencia n (%)	OR (IC_95%_)	p
Tratamiento empírico inicial						
	Meropenem	45 (42)	36 (42)	9 (39)	1,03 (0,43-2,5)	0,93
	Piperacilina-tazobactam	62 (57)	48 (57)	14 (61)	1,19 (0,47-3,18)	0,72
	Ceftriaxona	1 (1)	1 (1)	0	-	
	Vancomicina*	51 (47)	39 (46)	12 (52)	1,28(0,50-3,30)	0,76
Tratamiento definitivo						
	Meropenem	28 (26)	18 (21)	10 (43)	2,86 (0,94-8,29)	0,06
	Piperacilina-tazobactam	23 (21)	16 (19)	7 (3)	1,88 (0,63-5,32)	0,35
	Ceftriaxona	23 (21)	18 (21)	5 (22)	1,03 (0,31-3,10)	0,93
	Vancomicina	34 (32)	33 (39)	1 (4)	0,07 (0,01-0,43)	0,001

* Todos los pacientes recibieron un antibiótico betalactámico en el esquema empírico inicial. El 47% (51/108) recibieron un tratamiento combinado con vancomicina.

Las especies más frecuentemente identificadas fueron *S. mitis* y *S. oralis*, en 103 (95 %) episodios. En 4 (3,7 %) episodios el agente causal fue *S. salivarius* y, en uno (1 %), *S. sanguinis*. La sensibilidad de los estreptococos del grupo *viridans* a los antimicrobianos se presenta en el cuadro 4.

**Cuadro 4:** Sensibilidad a los antimicrobianos de microorganismos aislados (N = 108)

Variable		Total n (%)	Primer o único episodio n (%)	Recurrencia n (%)	OR (IC_95%_)	p
Sensibilidad a penicilina						
	Sensible	58 (54)	46 (54)	12 (52)	0,92 (0,36-2,32)	0,88
	Intermedia	31 (29)	24 (28)	7 (30)	1,11 (0,40-3,04)	0,84
	Resistente	11 (10)	11 (13)	-	-	
	Sin evaluar	8 (7)	5 (6)	3 (13)	2,4 (0,52-10,80)	0,24
Sensibilidad a ceftriaxona						
	Sensible	91 (84)	70 (82)	21 (91)	1,25 (0,47-10,60)	0,29
	Intermedia	9 (8)	7 (8)	2 (9)	1,06 (0,25-5,49)	0,90
	Resistente	7 (6)	7 (8)	0	-	
	Sin evaluar	3 (2)	3 (4)	0	-	
Sensibilidad a vancomicina						
	Sensible	106 (98)	84 (99)	22 (96)	0,26 (0,02-4,3)	0,31
	Intermedia	0	0	0	-	-
	Resistente	0	0	0	-	-
	Sin evaluar	2 (2)	1 (1)	1 (4)	3,81 (0,22-83,5)	0,31

Se clasificaron 85 (79 %) episodios como «primer o único episodio» y, 23 (21 %), como «recurrencias» de bacteriemia por estreptococos del grupo *viridans*. En el análisis univariado, tanto al comparar las características de los pacientes con un primer o único episodio de bacteriemia por estos estreptococos, como en las recurrencias, no se encontraron diferencias estadísticamente significativas respecto a la edad, el sexo, las enfermedades de base, el tratamiento empírico, ni la sensibilidad a los antibióticos de los microorganismos identificados (cuadros 1, 3 y 4). El uso de vancomicina en el tratamiento definitivo fue más frecuente en el primer o único episodio de bacteriemia.

Los siguientes factores se asociaron significativamente con la recurrencia de la bacteriemia:

1. 20 de 23 pacientes (87 %) presentaron recurrencia y 52 de 85 pacientes (61 %) no la presentaron;
2. la mayor mediana de días de tratamiento antibiótico previo [n = 9 vs. n = 3, p = < 0,01];
3. la gastroenteritis aguda como foco clínico de infección [n = 6 (26 %) vs. n = 8 (9 %), p = 0,01], y
4. la consolidación como etapa del tratamiento 11 de 23 pacientes (48 %) y 22 de 85 pacientes (26 %).

## Discusión

Los estreptococos del grupo *viridans* son un conjunto heterogéneo de microorganismos grampositivos, pertenecientes al género *Streptococcus*, que forman parte de la flora habitual de la cavidad oral, las vías aéreas superiores, el tubo digestivo y la mucosa genital ^6^. En los pacientes sin comorbilidades, las infecciones causadas por estos microorganismos son infrecuentes y no se asocian con gran mortalidad. Sin embargo, en individuos inmunocomprometidos, las infecciones por estas bacterias pueden manifestarse de manera grave y con alta tasa de morbimortalidad ^1^^,^^2^.

En los últimos años, se ha observado un aumento en la incidencia de infecciones por estos microorganismos, tanto en adultos como en niños, fenómeno que se atribuye a la intensificación de los esquemas quimioterapéuticos, al aumento de los trasplantes de precursores hematopoyéticos y a la mayor supervivencia de los pacientes con enfermedades oncohematológicas ^7^.

En Latinoamérica, en estudios realizados en Chile y Argentina, se identificaron a los estreptococos del grupo *viridans* como una causa frecuente de bacteriemia en niños con neutropenia febril, con un incremento sostenido en el tiempo ^8^^,^^9^. Estos hallazgos concuerdan con los observados en nuestra cohorte.

El aumento de estas infecciones se ha asociado principalmente con la presencia de mucositis inducida por quimioterapia intensiva, el uso de catéteres venosos centrales y la exposición prolongada a antibióticos de amplio espectro ^4^^,^^10^.

En concordancia con la literatura, en nuestro estudio predominaron los pacientes con leucemia mieloide aguda, neutropenia febril y antecedentes de tratamiento con citarabina, que son factores de riesgo ampliamente descritos para bacteriemia por estreptococos del grupo *viridans*^3^^,^^11^.

La mucositis también se reporta como un factor predisponente para la infección por estos microorganismos, con presencia en el 9 % de los episodios de esta serie, el cual es un porcentaje menor en comparación con el de otras series (8). Esta diferencia de porcentajes entre series podría explicarse por diferencias en la intensidad de los esquemas de quimioterapia, en los criterios de registro clínico o en las estrategias locales de prevención y manejo de la mucositis.

La mortalidad observada en nuestra cohorte fue del 7 %, valor comparable al reportado en otras series pediátricas ^3^^,^^8^, aunque debe interpretarse en el contexto de una población con enfermedad de base avanzada, dado que un porcentaje relevante de los pacientes se encontraba en recaída de su leucemia. Esto refuerza la idea de que la gravedad de la infección por estreptococos del grupo *viridans* está fuertemente condicionada por el estado del huésped más que por la virulencia intrínseca del microorganismo.

En relación con las especies aisladas, *S. mitis* y *S. oralis* fueron las predominantes, hallazgo congruente con lo descrito en series pediátricas y de adultos con neoplasias hematológicas ^8^^,^^12^. En un estudio realizado en México, *S. mitis* y *S. oralis* predominaron en los pacientes con cáncer, y se asociaron significativamente con neoplasias hematológicas y con el uso de antagonistas de la pirimidina, mientras que otros factores, como la diabetes, se asociaron con bacteriemias por estreptococos del grupo *viridans* no *mitis*^12^.

La inclusión específica de pacientes con leucemia en nuestra cohorte, así como la gran frecuencia de esquemas que incluyen citarabina, podrían explicar el marcado predominio observado de *S. mitis* y *S. oralis*, probablemente en relación con el daño de la mucosa y la translocación bacteriana.

Respecto a la sensibilidad a la penicilina, en nuestra serie, el 58 % de los aislamientos resultaron sensibles, valor intermedio en comparación con lo reportado en la literatura internacional, donde se describen tasas de resistencia que oscilan entre el 30 y el 70 % ^8^^,^^13^^,^^14^, y con los estudios locales que informan entre el 30 y el 60 % de cepas no sensibles ^15^.

La frecuencia de resistencia a los antimicrobianos es multifactorial. Las variaciones en el uso de antimicrobianos, particularmente en la profilaxis antibiótica y en el tratamiento empírico, así como por diferencias en la distribución de especies dentro del grupo de estreptococos *viridans* podrían explicar esta variabilidad.

El impacto clínico de la resistencia a la penicilina en las infecciones por estreptococos del grupo *viridans* continúa siendo controversial. Si bien algunos estudios la han asociado con mayor mortalidad ^16^^,^^17^, estos hallazgos no fueron confirmados en series posteriores de mayor tamaño ^14^^,^^18^, lo que sugiere que otros factores relacionados con el huésped y con el manejo clínico podrían desempeñar un rol más relevante en la evolución. En este sentido, Shelburne *et al*. desarrollaron un modelo predictivo de resistencia a los betalactámicos en pacientes adultos, con el cual identificaron que la exposición previa a antibióticos betalactámicos y la bacteriemia por estreptococos del grupo *viridans* durante la hospitalización son variables asociadas ^14^.

Si bien dicho modelo no fue validado de forma prospectiva ni en población pediátrica, estos hallazgos respaldan la hipótesis de que la presión antibiótica previa podría contribuir a la emergencia de cepas menos sensibles, más que la recurrencia en sí misma. Esta hipótesis podría explicar la ausencia de diferencias significativas en los perfiles de sensibilidad entre el primer episodio y las recurrencias observada en nuestra cohorte, aunque se requieren más estudios para explorar este tema.

En cuanto al tratamiento, las guías actuales no recomiendan el uso sistemático de vancomicina en pacientes con neutropenia febril ^19^^-^^21^. Si bien en algunos centros se propone su uso empírico en pacientes con mayor riesgo de bacteriemia por estreptococos del grupo *viridans*^22^, no se ha demostrado un beneficio en términos de la mortalidad. En nuestra serie, el mayor uso de vancomicina en el primer episodio podría relacionarse con la presentación clínica inicial y la sospecha de infección grave, más que con diferencias microbiológicas entre episodios.

La profilaxis antibiótica para infecciones por estreptococos del grupo *viridans* continúa siendo controvertido. Si bien en diversos estudios se ha demostrado que la profilaxis antibiótica en pacientes pediátricos con neutropenia reduce la incidencia de bacteriemia y de neutropenia febril, no se ha evidenciado un impacto en la mortalidad, y su uso se ha asociado con un aumento de la resistencia antimicrobiana ^23^^-^^26^. Hasta el momento, no existen estudios diseñados específicamente para evaluar la eficacia de la profilaxis antibiótica en la prevención de bacteriemias por estreptococos del grupo *viridans*, por lo que su uso debe evaluarse de manera individual, priorizando estrategias de uso racional de los antibióticos.

La recurrencia de bacteriemia por estreptococos del grupo *viridans* ha sido escasamente estudiada. En nuestra cohorte, los pacientes con recurrencia fueron aquellos que tuvieron mayor exposición previa a antibióticos y mayor duración del tratamiento antibiótico, hallazgos que sugieren que la presión antimicrobiana podría desempeñar un rol relevante. Asimismo, la asociación de la recurrencia con gastroenteritis aguda y con la etapa de consolidación podría reflejar un mayor compromiso de la barrera mucosa intestinal durante estos períodos del tratamiento, facilitando la translocación bacteriana. La ausencia de asociación estadística con algunas variables, como el uso previo de carbapenem, podría explicarse por el tamaño muestral limitado.

Las principales fortalezas de este estudio son el gran número de pacientes con bacteriemia por estreptococos del grupo *viridans* registrados en un corto periodo, y el registro metódico de los datos clínicos y microbiológicos en la historia clínica que permiten explorar la asociación entre las distintas variables y la recurrencia de la bacteriemia.

Sin embargo, se trata de un estudio realizado en un único centro y con un diseño retrospectivo, lo cual limita la generalización de los resultados y no permite evaluar la efectividad de los tratamientos instaurados. Por otra parte, el tamaño muestral relativamente pequeño no permitió desarrollar modelos multivariados, como análisis de regresión logística, para identificar factores asociados con la recurrencia. Además, la inclusión de pacientes con un único hemocultivo positivo podría haber hecho sobreestimar el número de episodios.

Futuros estudios prospectivos y multicéntricos permitirán profundizar en las características clínicas y microbiológicas de la bacteriemia por estreptococos del grupo *viridans* en pacientes pediátricos oncohematológicos, y contribuir a optimizar el diagnóstico y el tratamiento oportuno de esta infección.

En conclusión, en esta cohorte de niños con leucemia y bacteriemia por estreptococos del grupo *viridans*, predominó la leucemia mieloide aguda, con una sensibilidad a la penicilina del 58 % y una tasa de recurrencia del 21 %. La exposición previa a antibióticos, la diarrea como foco clínico y la etapa de consolidación, se asociaron con la recurrencia. Estos hallazgos aportan información relevante para la identificación de pacientes de mayor riesgo, y podrían contribuir al diseño de estrategias preventivas y terapéuticas más racionales en este grupo vulnerable.
